# Integrative taxonomy and mitogenome characterization of the root-knot nematode *Meloidogyne silvestris*

**DOI:** 10.1038/s41598-026-54669-9

**Published:** 2026-05-27

**Authors:** Justus Aisu, Gerrit Karssen, Daniel Apolônio Silva De Oliveira

**Affiliations:** 1 Nematology Research Unit, Department of Biology, Ghent University, Ledeganckstraat 35, Ghent, B-9000 Belgium; 2 National Plant Protection Organization the Netherlands (NPPO-NL), Geertjesweg 15, Wageningen, 6706 EA The Netherlands; 3 Geobotany and Botanical Garden, Martin Luther University Halle- Wittenberg, Halle (Saale), Germany; 4 German Centre of Integrative Biodiversity Research (iDiv) Halle-Jena- Leipzig, Leipzig, Germany

**Keywords:** *Meloidogyne silvestris*, Root-knot nematodes, Mitochondrial genome, Integrative taxonomy, Species delimitation, Diagnostic markers, Phylogenetics, Biological techniques, Computational biology and bioinformatics, Ecology, Ecology, Evolution, Genetics, Molecular biology, Plant sciences

## Abstract

Root-knot nematodes (*Meloidogyne* spp.) are among the most damaging plant-parasitic nematodes, yet taxonomic uncertainties often complicate diagnostics and management. Here, we examined *Meloidogyne silvestris* populations from the Netherlands using an integrative approach combining morphology, isoenzyme profiles, and molecular markers. Morphological and morphometric traits, together with isozyme profiles, were highly consistent between the two Dutch populations and were complemented by near-identical 18S and 28S rDNA sequences, identical *cox1* and *cox2* barcodes, and a conserved mitochondrial genome architecture. In comparison with the original species description, these data support their identification as *M. silvestris*. We also present a nearly complete mitochondrial genome of *M. silvestris*, which expands genomic resources for root-knot nematodes. These findings highlight the value of integrative datasets for robust species delimitation and provide a framework for future studies on population structure, evolutionary dynamics, and diagnostic marker development within the genus.

**Supplementary Information:**

The online version contains supplementary material available at 10.1038/s41598-026-54669-9.

## Introduction

Root-knot nematodes (RKNs) of the genus *Meloidogyne* are the most important plant-parasitic nematodes in agriculture, posing a major threat to global food security by affecting a wide range of economically important crops^1^. To date, more than 105 validated *Meloidogyne* species have been described. Among these, *M. incognita*, *M. javanica*, *M. arenaria*, and *M. hapla* are considered the most widespread and damaging^2^. These species parasitize a broad range of monocot and dicot hosts, including both herbaceous and woody plants^3^.

In Europe, several *Meloidogyne* species; including *M. ardenensis*, *M. arenaria*, *M. baetica*, *M. hispanica*, *M. javanica*, *M. lusitanica*, and *M. hapla* have been documented parasitizing woody plants across boreal, temperate, and mediterranean regions^4^. Some of these, such as *M. baetica*, appear largely restricted to woody hosts within their native ranges^5^. A more recently described member of this group is *Meloidogyne silvestris*, a species showing similar preference for woody plant hosts.

*M. silvestris* was first described from *Ilex aquifolium* host plant in Spain^6^. Our study documents its first occurrence beyond the type locality, with populations identified in two Dutch municipalities (Hilversum and Bennekom). According to the European and Mediterranean Plant Protection Organization (EPPO) quick scan report, these populations exhibited an extended host range, parasitizing *Ulmus hollandica*, *U. glabra*, and alongside the original *I. aquifolium* host plants^7^.

The systematics and phylogeny of *Meloidogyne* have undergone major revision over the last two decades, driven largely by the integration of molecular data. Early phylogenetic analyses based on the internal transcribed spacer (ITS) region revealed extensive cryptic diversity and incongruence with traditional morphology-based classifications^8^. More recently, comprehensive multigene and phylogenomic approaches have provided a robust framework for understanding evolutionary relationships within the genus, revealing multiple deeply divergent lineages and frequent cases of morphological convergence^9,10^.

While morphological identification remains central to nematode taxonomy, it demands significant expertise and is frequently complicated by phenotypic plasticity, overlapping morphological and morphometric traits among closely related species^11–13^. Isozyme methods such as malate dehydrogenase (Mdh; EC 1.1.1.37) and esterase (Est; EC 3.1.1.1.), while valuable for specific diagnostic applications, are limited to particular developmental stages typically young adult females and may yield inconsistent results across laboratories due to variation in protein extraction, sample storage and electrophoretic conditions^14^.

Molecular markers have greatly enhanced the precision and reliability of nematode identification. Nuclear ribosomal regions, including 18S rDNA, 28S rDNA, and the internal transcribed spacer (ITS), are widely used for species barcoding and community-level analyses^15,16^. However, these markers also have limitations: the 18S gene is often too conserved to distinguish closely related species, while ITS and 28S regions may exhibit high intraspecific variability^17,18^.

Mitochondrial DNA (mtDNA) markers, particularly protein-coding genes (PCGs) such as *cox1*,* cox2*, and *cytb*, have become increasingly important in diagnostics and phylogenetic studies^19,20^. Advances in high-throughput sequencing have further expanded their utility in nematode diagnostics, phylogenomics, and comparative genomics^21–23^. However, mitochondrial markers are not without caveats, as maternal inheritance patterns and potential introgression events can obscure phylogenetic interpretations, necessitating careful consideration in taxonomic assessments^24,25^.

Given the respective strengths and limitations of each identification method, an integrative approach combining morphological, biochemical, and molecular data is increasingly recognized as essential for robust nematode diagnostics and taxonomy. Here, we applied such framework with three objectives: (1) report for the first time the occurrence of *M. silvestris* outside its type locality; (2) to expand the species phenotypic and molecular characterization through morphometrics, isozymes and multi-locus DNA analyses, and (3) present its first near-complete mitogenome for characterization and evolutionary insights.

## Materials & methods

### Field sampling and nematode extraction

Field sampling was conducted in October 2022 at two locations in the Netherlands: Hilversum (52°23’53.9"N, 5°18’42.3"E) and Bennekom (51°59’38.1"N, 5°40’34.1"E). The Hilversum site was targeted following the EPPO quick scan that reported *M. silvestris* in the area, whereas the Bennekom site was selected based on the presence of host plants showing clear root-knot nematode feeding symptoms. At the Hilversum site, root samples were collected from *Ilex aquifolium* (common holly), while *Euonymus fortunei* (wintercreeper) roots were sampled in Bennekom.

Plant identification was carried out by a senior botanist at the Netherlands National Plant Protection Organization. The sampled species are listed as Least Concern on the International Union for Conservation of Nature (IUCN) Red List and are not included in the Convention on International Trade in Endangered Species of Wild Fauna and Flora (CITES); therefore, no special permits or licenses were required for sampling.

Fine root segments (< 1 cm length) were processed using the mist chamber extraction method^26^ to isolate second-stage juveniles (J2s) and males of *M. silvestris*. Adult females were carefully extracted from root galls using fine dissection tools under a Leica stereomicroscope at 40× magnification. For diagnostic purposes, only J2s and females were used for primary taxonomic identification, as males of *Meloidogyne* species typically provide limited taxonomic information. Nevertheless, to complement our morphometric analyses, adult males were also examined.

### Preparation for morphology, morphometry and biochemical analysis

Freshly extracted J2s were individually selected using a nematode picking needle under a Leica stereomicroscope and temporarily mounted for examination under a Leica DM2500 differential interference contrast (DIC) microscope (Leica Microsystems GmbH, Wetzlar, Germany) at 50×, 100×, 200×, 400×, 1000× magnification. High-resolution images were captured using a Leica DFC450 camera coupled with IMAGIC-IMS Professional Imaging software v21H1 (Bildverarbeitung AG, Glattbrugg, Zurich, Switzerland).

From each population, 20 J2 individuals were analyzed, with 14 primary morphological measurements and 5 derived morphometric indices recorded per specimen (19 variables in total). Additionally, 12 adult males were analyzed, with 15 primary morphological measurements and 5 derived morphometric indices recorded per individual (20 variables in total; Table 1). Morphologically characterized specimens were preserved at -20 °C for subsequent molecular analysis. For perineal pattern preparation, the anterior region (head and neck) of young females was removed, and the posterior end was cleared of residual body tissues to isolate the perineal pattern. Temporary slides were then prepared for observation.

**Table 1:** Morphometric measurements of adult males and second-stage juveniles (J2s) of *M. silvestris* populations from the Netherlands, compared with data from the original type population. All measurements are in *µ*m.

Character	The Netherlands (Hilversum)		The Netherlands (Bennekom)		Spain	
	Males	Juveniles	Males	Juveniles	Males	Juveniles
	Mean ± SD		Mean ± SD		Mean ± SD	
n	12	20	12	20	12	20
L	1610.97 ± 152.8 (1436.89-1894.2)	469.76 ± 33.95 (399.14-522.26)	1538.47 ± 200.6 (1298.88-1992.97)	438.6 ± 33.67 (390.96-528.85)	1864 ± 184 (1565–2141)	506 ± 21·3 (465–539)
Max body diam.	41.94 ± 2.65 (35.18–44.65)	24.83 ± 2.43 (17.59–29.77)	41.6 ± 2 (37.88–44.65)	19.41 ± 2.47 (15.04–24.61)	38 ± 3·2 (30–42)	16·5 ± 0·8 (15·3–18)
Pharynx (to cardia)	154.69 ± 11.56 (135.73-172.75)	97.35 ± 4.9 (88.06-110.08)	161.57 ± 20.83 (126.59–206.4)	94.74 ± 5.12 (85.31-101.82)	153 ± 12·6 (135–170)	99 ± 6·6 (89–108)
Head to hemizonid	153.89 ± 13.8 (130.25-168.63)	86.41 ± 20.75 (0-101.82)	153.89 ± 13.8 (130.25-168.63)	84.82 ± 9.07 (63.3-95.63)	–	–
Head end to EP	160.81 ± 14.68 (137.1-178.23)	94.46 ± 5.88 (81.18-108.02)	142.65 ± 45.35 (0-191.26)	85.59 ± 9.63 (64.67–97.7)	158 ± 8·8 (145–176)	94 ± 4·9 (84–104)
Stylet	25.58 ± 1.01 (23.64–27.52)	14.52 ± 0.68 (13.76–15.14)	25.11 ± 0.82 (24.77–27.52)	15.14 ± 0.62 (13.76–16.51)	25·6 ± 1·1 (24–27·3)	12·8 ± 0·5 (12·3–14·3)
Stylet conus	13.22 ± 1.35 (10.01–15.14)	7.74 ± 0.72 (6.19–8.26)	13.53 ± 0.76 (12.38–15.14)	9.56 ± 0.3 (8.26–9.63)	12 ± 0·9 (10·7–14)	6·5 ± 0·4 (6·0–7·7)
Knob width	4.93 ± 0.68 (4.13–5.5)	1.72 ± 0.6 (1.38–2.75)	4.59 ± 0.65 (4.13–5.5)	2.61 ± 0.41 (1.38–2.75)	5·0 ± 0·4 (4·5–5·5)	2·4 ± 0·3 (2·0–2·7)
Head to center of Median bulb	97.81 ± 3.1 (90.82-101.82)	67.29 ± 5.59 (60.54–88.06)	93.11 ± 4.02 (82.56-100.45)	62.54 ± 4.21 (55.04–70.18)	109 ± 6·1 (97–117)	66 ± 5·2 (53–72)
D.G.O	5.05 ± 0.65 (4.13–5.5)	3.23 ± 0.66 (2.75–4.13)	4.24 ± 1.43 (0-5.5)	4.2 ± 0.53 (2.75–5.5)	6·0 ± 0·5 (5·3–6·7)	3·0 ± 0·6 (2·0–4·0)
Tail length	9.75 ± 0.68 (8.26–11.01)	42.24 ± 3.35 (35.78–49.54)	9.06 ± 1.43 (6.88–12.38)	40.48 ± 1.48 (38.53–43.9)	10·5 ± 1·2 (8·5–12·5)	44 ± 2·8 (37·3–48·7)
Anal body diameter	21.44 ± 1.31 (20.64–24.77)	14.38 ± 0.68 (13.76–15.14)	19.58 ± 0.79 (17.89–20.64)	11.76 ± 1.11 (9.63–13.76)	25 ± 2·6 (20–29·5)	11 ± 1·0 (9·3–13)
Spicules	37.38 ± 0.76 (35.78–38.53)	-	37.61 ± 2.88 (30.27–41.28)	–	33·3 ± 2·5 (28·7–38)	-
Gubernaculum	9.17 ± 0.65 (8.26–9.63)	-	9.06 ± 0.88 (6.88–9.63)	–	9·7 ± 1·3 (6·7–11·3)	-
a (Body length/ Body width)	38.53 ± 4.04 (33.19–46.47)	19.11 ± 2.41 (14.75–25.92)	36.91 ± 3.78 (32-46.03)	22.91 ± 3.04 (15.89–31.55)	49·2 ± 4·5 (41·9–56·4)	30·8 ± 1·5 (27·9–32·9)
b (Body length / Pharynx length	10.44 ± 0.92 (8.73–11.66)	4.84 ± 0.44 (3.92–5.42)	9.65 ± 1.55 (7.55–12.39)	4.63 ± 0.31 (4.13–5.45)	12·1 ± 1·0 (10·6–13·5)	5·2 ± 0·2 (4·7–5·5)
c (Body length / Tail length)	165.66 ± 15.24 (141.35-195.81)	11.18 ± 1.12 (9.06–13.34)	174.21 ± 36.22 (111.44–241.4)	10.85 ± 0.89 (9.29–12.4)	178·4 ± 28·4 (146·7–234·1)	11·5 ± 0·7 (10·4–13·7)
c’ (Tail length / Anal body diameter)	0.46 ± 0.03 (0.4–0.5)	2.94 ± 0.26 (2.55–3.4)	0.46 ± 0.07 (0.38–0.6)	3.47 ± 0.3 (2.9–4.14)	0·4 ± 0·1 (0·3–0·5)	4·0 ± 0·4 (3·3–4·7)
m (stylet conus/ stylet length)	0.52 ± 0.04 (0.41–0.56)	0.53 ± 0.05 (0.41–0.6)	0.54 ± 0.03 (0.5–0.58)	0.63 ± 0.02 (0.58–0.7)		
Tail hyaline portion	–	14.17 ± 1.31 (11.01–15.14)	–	13.76 ± 1.38 (11.01–15.14)	–	15·3 ± 1·2 (14–18·7)
Head diameter	12.5 ± 0.38 (12.38–13.76)	4.95 ± 0.67 (4.13–5.5)	13.3 ± 0.86 (12.38–15.14)	5.5 ± 0 46(5.1–5.6)	–	–

### Biochemical analysis

Isozyme analysis targeting Mdh; EC 1.1.1.37 and Est; EC 3.1.1.1, was conducted on all populations. The methodology for the Hilversum population followed Karssen et al., 1995^27^. Briefly, ten young females were isolated and maintained in 0.9% NaCl. They were then desalted in distilled water on an ice bath for no longer than 5 min and individually macerated in 0.6 µL extraction buffer using a metal rod. The homogenized protein lysate was separated via polyacrylamide gel on a PhastGel system (Cytiva, Uppsala, Sweden).

For the Bennekom population, the same procedure was followed using ten young females, except that Novex™ WedgeWell™ Tris-Glycine Mini Gels (Invitrogen™, Thermo Fisher Scientific, Carlsbad, CA, USA) were used instead of the PhastGel system. In both cases, the gels were stained for Mdh and Est activity for 30 and 60 min, respectively. After staining, gels were rinsed with distilled water and fixed in a solution of 10% glycerol, 10% acetic acid, and distilled water. *M. javanica* was included as a reference at the center of the gels.

### DNA extraction and Illumina sequencing

ClearDetection kit (Wageningen, The Netherlands) was used for DNA extraction of nematodes from both populations. Briefly, samples were lysed with proteinase K and Dithiothreitol (DTT) in 50 µL of 2x extraction buffer, incubated at 65 °C for 30 min with shaking at 800 rpm, followed by incubation at 95 °C for 5 min. The condensate was briefly centrifuged for (30 s at 1500 × g) and stored at -20 °C.

About 30 µL of extracted genomic DNA (gDNA) from each sample was sent to GenomeScan (Leiden, The Netherlands) for 150 bp paired-end sequencing using Illumina technology. Prior to library preparation, the gDNA was fragmented with a Bioruptor Pico (Diagenode, Belgium). Library preparation was conducted using the NEBNext Ultra II DNA Library Prep Kit (New England Biolabs, Ipswich, England), following the manufacturer’s instructions.

DNA quality and concentration before and after library preparation were assessed with 2100 Bioanalyzer, developed by Agilent, California, USA. Commercially available Cat (*Felis silvestris catus L*) was included as Positive process control (PPC) to detect possible contamination during the library prep.

### Sequence assembly, annotation and barcoding

Raw Illumina reads were processed using CLC Genomics Workbench v23.0.4 (Qiagen, USA) with an automated pipeline^28^. Contigs were quality-filtered and visualized using Krona^29^.

For ribosomal DNA (rDNA), only 18S and 28S genes were retained for downstream analysis. Fragmented rDNA sequences were *de novo* assembled in Geneious Prime v2020.0.5 (Biomatters Ltd., Auckland, New Zealand) (http://www.geneious.com/) to generate complete contigs when necessary.

Putative mtDNA contigs were identified via BLASTn against a custom database and annotated using MITOS and MITOS v2^30,31^. PCGs were manually curated for start/stop codons. Near complete mitogenome was plotted using OGDraw^32^.and nucleotide composition was analysed with strand asymmetry calculated as:

$${\text{AT skew}}={\text{ }}({\mathrm{A}}\, - \,{\mathrm{T}})/({\mathrm{A}}\,+\,{\mathrm{T}})$$

$${\text{GC skew}}={\text{ }}({\mathrm{G}}\, - \,{\mathrm{C}})/({\mathrm{G}}\,+\,{\mathrm{C}})$$

rDNA and mtDNA barcodes were generated computationally (in silico) in Geneious Prime using primer sets (Table 2), and alignments and similarity estimates were computed with MAFFT v7.490^33^. Secondary structures of predicted tRNAs were visualized using R2DT framework^34^.

**Table 2:** Primer names and corresponding sequences used in the in-silico analyses.

Primer name	Primer sequence 5′–3′	Target gene	References
COX1F	ATCCTCCTTTGATGATTGATGG	*Cox1*	^45^
COX1R	AACTCAATAAAGAACCAATAGAAG	*Cox1*	^45^
COX2F	TTGAATTTAAGTGTTGTTTATTAC	*Cox2*	^45^
COX2R	GATTAATACCACAAATCTCTGAAC	*Cox2*	^45^
COX3F	TTTTGCTGAGGATTAATAGG	*Cox3*	^45^
COX3R	TAAACTTCCATAAATACCATCAC	*Cox3*	^45^
D2A	ACAAGTACCGTGAGGGAAAGTT	28S rDNA	^60^
D3B	TCGGAAGGAACCAGCTACTA	28S rDNA	^60^
1813 F	CTGCGTGAGAGGTGAAAT	18S rDNA	^61^
1912R	TTTACGGTCAGAACTAGGG	18S rDNA	^61^
988 F	CTCAAAGATTAAGCCATGC	18S rDNA	^61^
2646R	GCTACCTTGTTACGACTTTT	18S rDNA	^61^
531	CTTCGCAATGATAGGAAGAGCC	18S rDNA	^62^
527	CTAAGGAGTGTGTAACAACTCACC	18S rDNA	^62^

### Phylogenetic and mitogenomic analysis

All phylogenetic trees were generated using Maximum Likelihood (ML) in MEGA v11.0.8^35^ with 1000 bootstrap replicates^36^. Nuclear rDNA phylogenies were inferred from 18S and D2–D3 28S rDNA alignments (2,105 bp and 1,106 bp, respectively). The alignment length reflects sequence length variation among taxa and gaps introduced during alignment. The best-fit substitution models, selected based on the Bayesian Information Criterion (BIC), were GTR + G+I for both the 18 S and 28S rDNA datasets. The generated trees from 18S rDNA dataset (48 *Meloidogyne* sequences) and 28S rDNA dataset (44 *Meloidogyne* sequences) were both rooted with *Pratylenchus penetrans* and *Pratylenchus vulnus.* GenBank accession numbers of gene sequences from *Meloidogyne* species used in the phylogenetic analysis are provided in Table S1.

For mitogenomic reconstruction, amino acid sequences of 12 mitochondrial PCGs from 33 nematodes (Chromadorea and Enoplea) were aligned using MAFFT in MEGA and concatenated. Poorly conserved or uninformative regions were removed with Gblocks^37^, resulting in a final alignment of 1,284 aa. The best-fit substitution model, LG + G+I + F, was selected based on the BIC. The phylogeny was rooted using two arthropod outgroups: *Limulus polyphemus* and *Lithobius forficatus*. Mitochondrial synteny was analyzed across nematode taxa (including *M. silvestris*) to identify gene order rearrangements. GenBank accession numbers of mitochondrial gene sequences used in these analyses are provided in Table S2.

## Results

### Morphology, morphometry and biochemical analysis

Females and J2s of *M. silvestris* from both populations exhibited morphological and morphometric uniformity to the original description, with slight variation in J2s body length, maximum body diameter, “a” ratio and stylet length. Among all measured traits, body length showed the highest variability (Table 1). Perineal patterns were similar between the two examined Dutch populations and consistent with the original description (Fig. S1d).

**Fig. 1:**
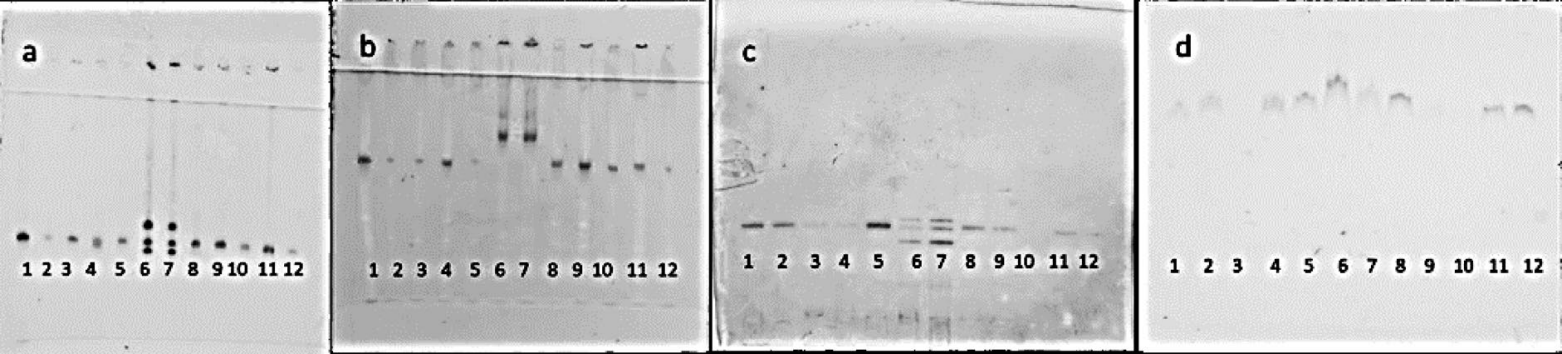
Esterase (**a** and **c**) and malate dehydrogenase (**b** and **d**) electrophoresis staining patterns of protein homogenates from young, egg-laying females of *Meloidogyne* spp. Lane 6 and 7 represent *Meloidogyne javanica* reference phenotypes, lane 1–5 and 8–12 represent *M. silvestris* phenotypes. a and b represent phenotypes form Hilversum population while c and d represent phenotypes from Bennekom population.

Biochemical profiles were also similar with the original species description^6^. *M. silvestris* from both populations showed a single A1 esterase (Est) band and an N1c malate dehydrogenase (Mdh) phenotype. Reference species *M. javanica* exhibited J3 Est and N1 Mdh phenotypes^38^(Fig. 1).

### DNA extraction for Illumina and de novo assembly

Measured DNA concentrations ranged in each sample ranged from 1.52 to 2.06 ng/µL. Illumina sequencing yielded a total output of 2Gb raw data and approximately 5.2 to 60.4 million reads per sample, resulting in 5,372 to 130,950 assembled contigs. The majority of assembled contigs did not produce BLASTn hits or aligned primarily to bacterial and fungal sequences. However, BLASTn searches against a locally installed NCBI nr/nt database produced 5–23 ribosomal DNA (rDNA) contigs and 2–6 mitochondrial DNA (mtDNA) contigs per sample.

### Ribosomal and mitochondrial DNA Sequences

From both Dutch populations, a total of eleven annotated rDNA contigs were obtained. For the 18S rDNA region, both partial (< 1000 bp) and complete (1730 bp) barcode sequences were retrieved. 18S rDNA contigs from the two Dutch populations showed 99.9% similarity to each other and ~ 99.36% similarity to the Spanish population. For 28S rDNA (768 bp), the D2–D3 region showed 99.7% similarity within Dutch populations and ~ 99.35% similarity to the Spanish population. Barcode sequences of 450 bp (*cox1*) and 432 bp (*cox2*) were obtained, with representative alignments showing 100% identity between the two populations (Fig. S2 c-d).

### Phylogenetic and mitogenomic analysis

**Fig. 2:**
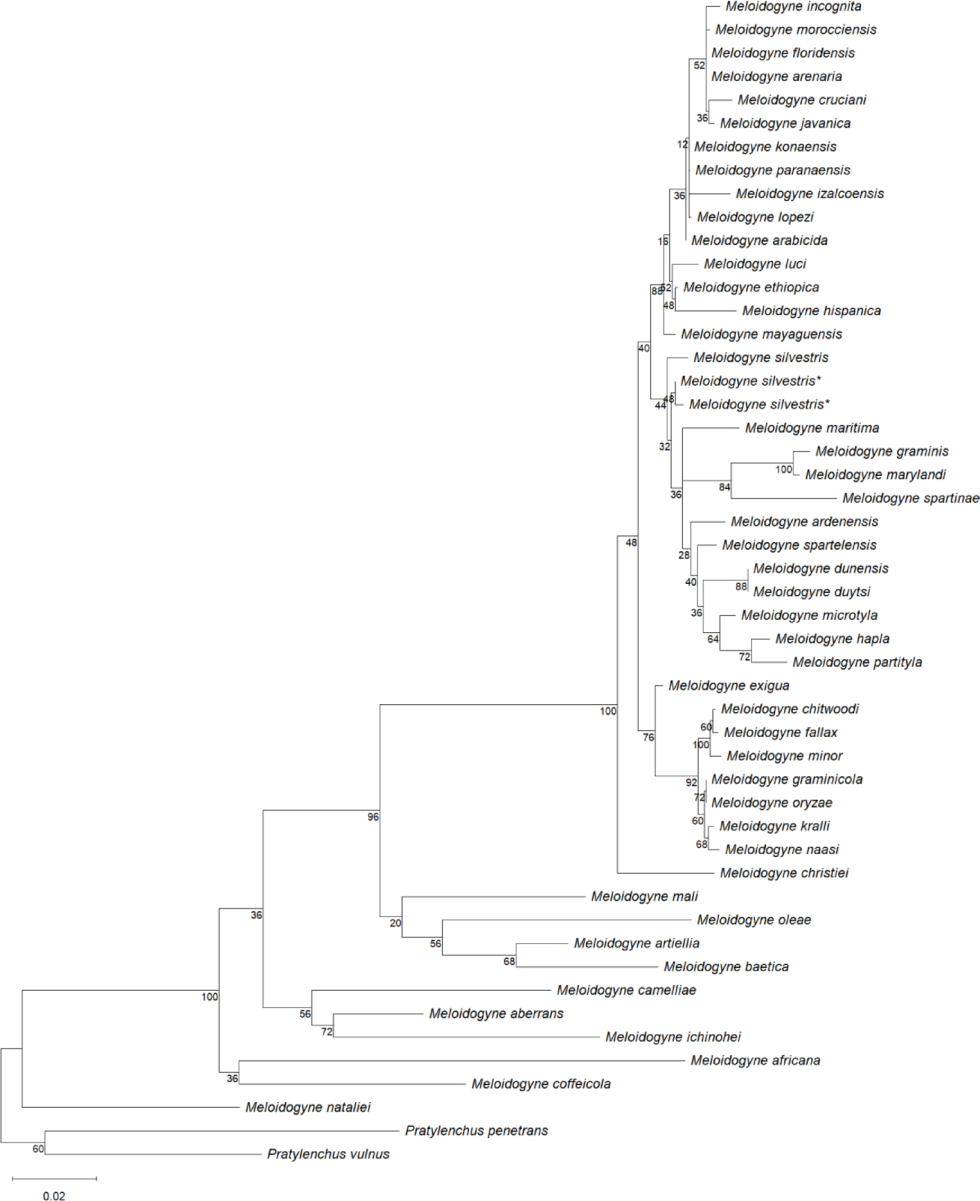
Maximum Likelihood tree based on alignments of 18S rDNA sequences of *M. silvestris* and other root-knot nematodes. Bootstrap values are shown at the corresponding nodes. Newly obtained sequences from both populations are marked with an asterisk (*).

**Fig. 3:**
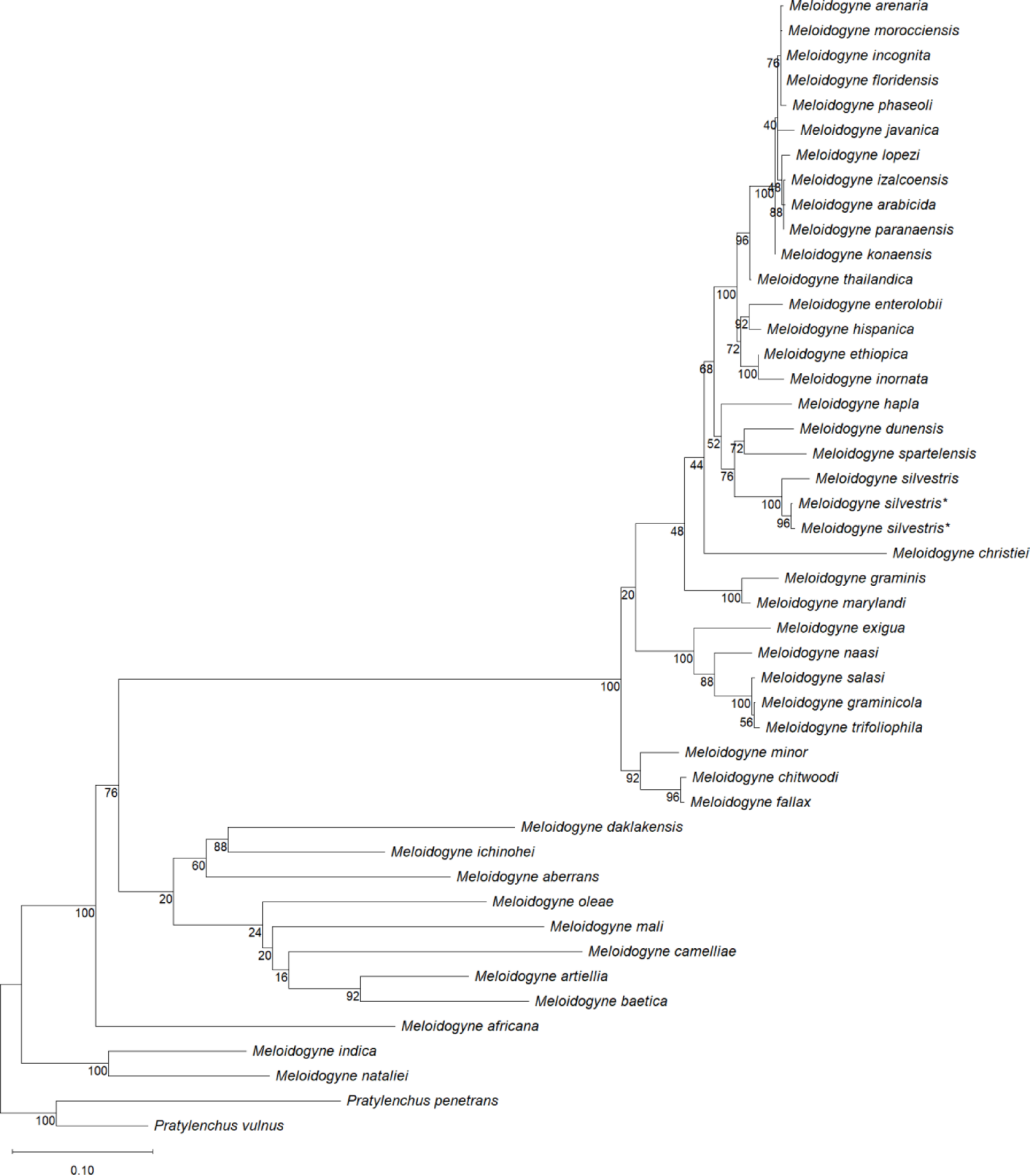
Maximum Likelihood tree based on alignments of D2–D3 28S rDNA sequences of *M. silvestris* and other root-knot nematodes. Bootstrap values are shown at the corresponding nodes. Newly obtained sequences from both populations are marked with an asterisk (*).

Phylogenetic analysis of 18S rDNA placed *M. silvestris* sequences from both sampling sites in a clade with conspecifics from the type locality, albeit with low bootstrap support (44%). This clade also included *M. ardenensis*, *M. dunensis*,* M. dutysi*,* M. graminis*,* M. hapla*, *M. maritima*, *M. marylandi*,* M. microtyla*, *M. partityla*,* M. spartelensis* and *M. spartinae* (Fig. 2). For 28S rDNA, sequences of *M. silvestris* from both sampling sites and the type locality formed a highly supported monophyletic clade (100% bootstrap). This clade also showed a sister-group relationship with *M. dunensis* and *M. spartelensis* though with moderate support (76% bootstrap) (Fig. 3).

**Fig. 4:**
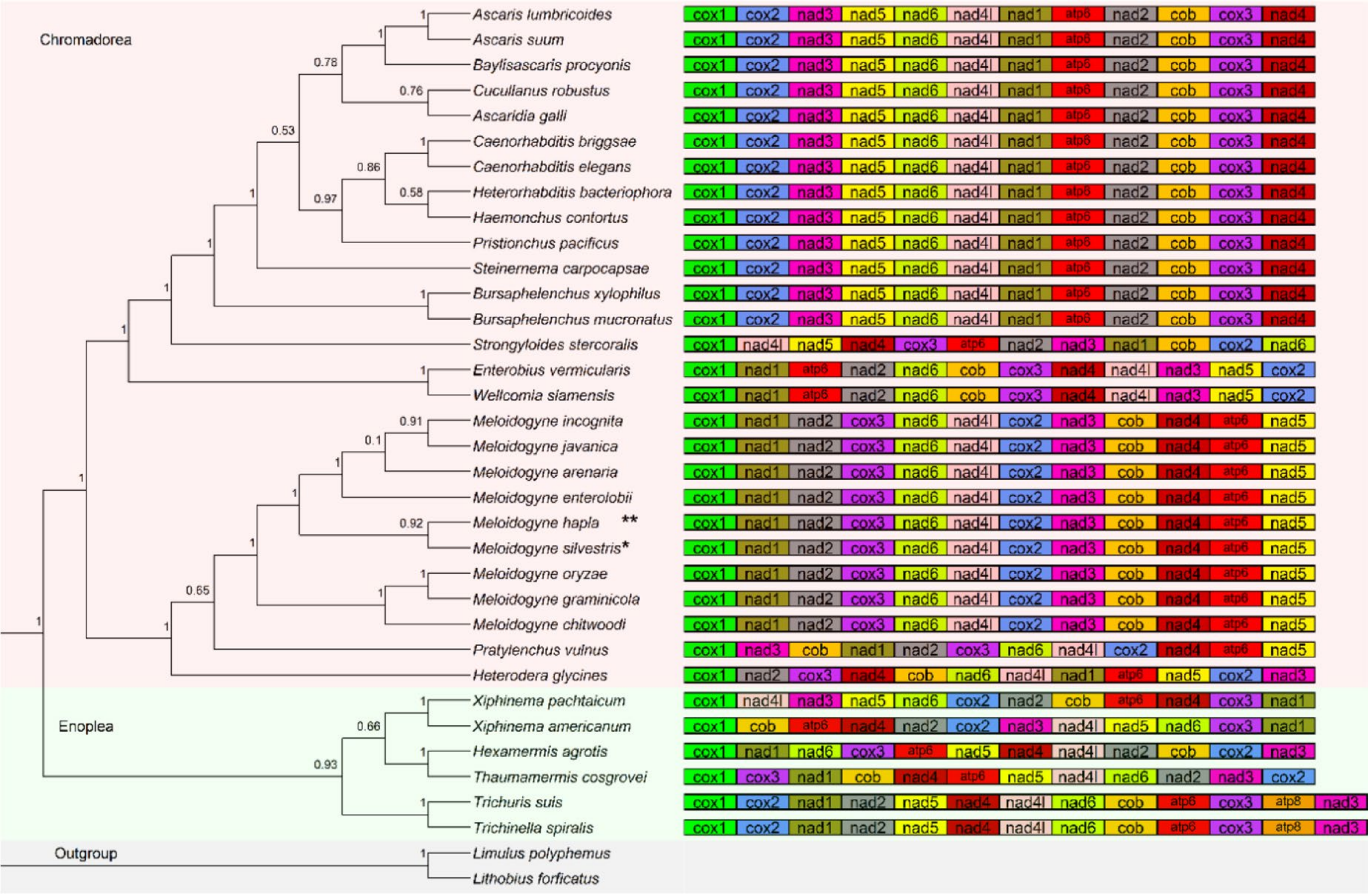
(Left) Maximum Likelihood mitogenomic tree inferred from amino acid sequences of 12 concatenated protein-coding genes. The newly obtained *M. silvestris* mitochondrial sequence is marked with a single asterisk (*) and unpublished sequence is marked with double asterisk (**). (Right) Linearized mitochondrial protein-coding genes (PCGs: *cox1*-*3* (cytochrome c oxidase subunits 1-3); *cob* (cytochrome b); *nad1-6* (NADH dehydrogenase subunits 1-6); *nad4L* (NADH dehydrogenase subunit 4 L); *atp6* (ATP synthase subunit 6) arrangement patterns of *M. silvestris* and other related species. *atp8* (ATP synthase subunit 8, present in some taxa; *Trichuris suis* and *Trichinella spiralis*)).

The amino acid ML mitogenomic tree grouped *M. silvestris* with *M. hapla*, while tropical (*M. incognita*,* M. javanica*,* M. arenaria*,* M. enterolobii*) and temperate (*M. oryzae*,* M. graminicola*,* M. chitwoodi*) RKNs formed two distinct, well-supported subclades (bootstrap support > 90%). Notably, the tree also showed that *P. vulnus* clustered more closely with *Meloidogyne* spp. than with *H. glycines* (moderate bootstrap support; Fig. 4, left). Comparative synteny analysis showed that the PCG order in *M. silvestris* was conserved across other *Meloidogyne* species and shared more similarity (four genes at the same position) with *P. vulnus* than with *H. glycines* (one gene at the same position; Fig. 4, right).

### Mitochondrial genome of M. silvestris

**Fig. 5:**
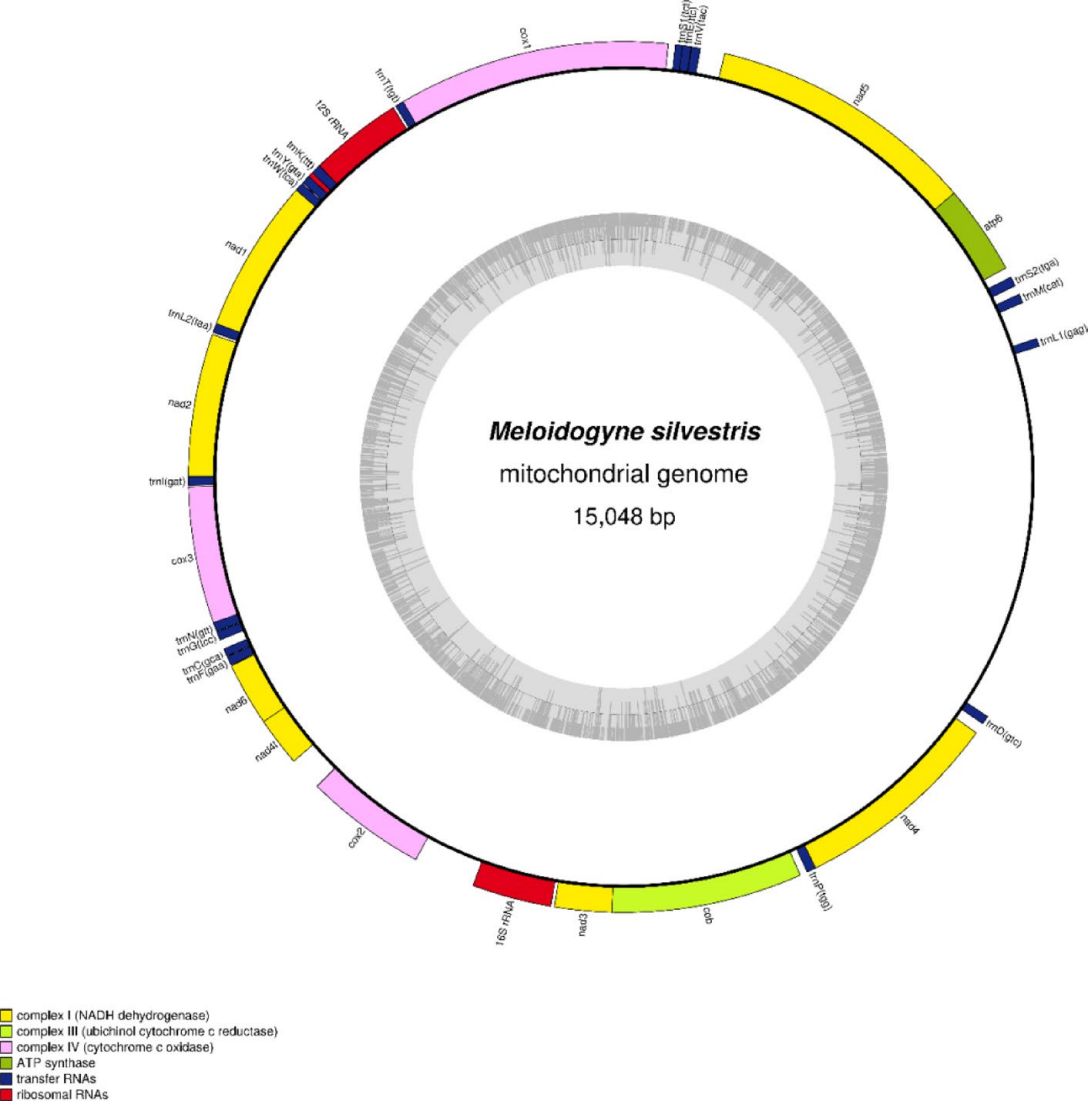
Gene arrangement in the 15,048 bp mitochondrial genome of *M. silvestris* obtained from specimen F6110-C_3697994. Functional gene categories are indicated by different colours as shown in the legend. The inner dark grey histogram displays the GC content across the genome, while the mid-grey line marks the 50% GC threshold.

The nearly complete mitochondrial genome of *M. silvestris* was assembled as a circular DNA molecule of 15,048 bp (Fig. 5). Its nucleotide composition was: A = 27.3%, T = 49.3%, C = 6.3%, G = 17.1% resulting in a strong A + T bias of 76.6%. The AT skew and GC skew were − 0.2866 and 0.4623, respectively.

Twelve protein-coding genes (PCGs) were annotated, including: *cox1*,* cox2*,* cox3* (cytochrome c oxidase subunits), *cob* (cytochrome b) *nad1-6*,* nad4L* (NADH dehydrogenase subunits) and *atp6* (ATP synthase subunit) comprising 10,053 bp residues. The longest genes were *cox1* (1525 bp) and *nad5* (1533 bp); the shortest were *nad4L* (176 bp) and *nad3* (324 bp) (Table 3).

**Table 3:** Mitochondrial genome organization of *M. silvestris*.

Name	Type	Minimum	Maximum	Length	Start codon	Stop codon	Direction
trnD(gtc)	tRNA	13,604	13,656	53			Forward
nad4	gene	12,335	13,546	1212	ATT	TAA	Forward
trnP(tgg)	tRNA	12,289	12,342	54			Forward
cob gene	gene	11,205	12,287	1083	ATT	TAA	Forward
nad3	gene	10,900	11,223	324	ATT	TAG	Forward
16 S rRNA	rRNA	10,436	10,903	468			Forward
cox2	gene	9367	10,055	689	ATT	TAA	Forward
nad4l	gene	8992	9167	176	ATT	TAA	Forward
nad6	gene	8604	8990	387	ATT	TAA	Forward
trnF(gaa)	tRNA	8541	8594	54			Forward
trnC(gca)	tRNA	8484	8540	57			Forward
trnG(tcc)	tRNA	8392	8444	53			Forward
trnN(gtt)	tRNA	8338	8391	54			Forward
cox3	gene	7576	8352	777	ATA	TAA	Forward
trnl(gat)	tRNA	7519	7571	53			Forward
nad2	gene	6715	7533	819	ATT	TAA	Forward
trnL2(taa)	tRNA	6660	6714	55			Forward
nad1	gene	5819	6633	815	TTG	TAG	Forward
trnW(tca)	tRNA	5750	5802	53			Forward
trnY(gta)	tRNA	5696	5749	54			Forward
trnK(ttt)	tRNA	5615	5672	58			Forward
12 S rRNA	rRNA	5121	5523	403			Forward
trnT(tgt)	tRNA	5059	5115	57			Forward
cox1 gene	gene	3515	5039	1525	ATT	TAA	Forward
trnS1(tct)	tRNA	3446	3502	57			Forward
trnE(ttc)	tRNA	3387	3440	54			Forward
trnV(tac)	tRNA	3331	3385	55			Forward
nad5	gene	1672	3204	1533	ATT	TAA	Forward
atp6	gene	1299	1703	405	ATG	TAG	Forward
trnS2(tga)	tRNA	1090	1148	59			Forward
trnM(cat)	tRNA	985	1039	55			Forward
trnL1(gag)	tRNA	725	777	53			Forward

Two gene overlaps were observed: 31 bp between *nad5* and *atp6*, 18 bp between *nad3* and *cob*. The longest intergenic spacer (382 bp) was located between *cox2* and 16S rRNA gene. Eighteen tRNA genes were identified ranging from 53 bp to 59 bp, all encoded in the same orientation as PCGs (Fig. 5). The predicted secondary structures of these tRNAs are provided in Fig. S3. Four tRNAs (*trnA*,* trnH*,* trnQ*, and *trnR*) were not recovered. The 16S rRNA gene (*rrnL*, 468 bp) was located between *cox2* and *nad3*, while the 12S rRNA gene (*rrnS*, 403 bp) was found between *cox1* and *nad1*, both showing similar nucleotide composition. A long non-coding region (2118 bp) was observed between *trnD* and *trnL1*.

## Discussion

Our combined morphological, biochemical, and molecular analyses provide a strong support that geographically separated populations of *M. silvestris* are conspecific, consistent with the original type population from Spain^6^. This is further supported by the consistently high sequence similarity observed across all markers.

Females and J2s from both Bennekom and Hilversum populations were largely morphologically uniform to the original description, with only slight variations observed in J2s body length, maximum body diameter, “a” ratio and stylet length. These levels of intraspecific variation are comparable to those reported in previous studies^39,40^. Isoenzyme analyses further supported species identity: both populations exhibited an A1 esterase (Est) band and an N1c malate dehydrogenase (Mdh) phenotype^41^, clearly distinct from reference species *M. javanica*.

ML phylogenetic trees based on 18S and 28S rDNA sequences complemented the phenotypic findings and were congruent with previously published maximum parsimony (MP) analyses^6,42^. As expected for conserved markers, 18S rDNA-based topologies exhibited limited species-level resolution, reflected in low bootstrap support for *M. silvestris*. By contrast, the D2–D3 region of 28S rDNA, which contains hypervariable domains, provided stronger phylogenetic signal, yielding a monophyletic *M. silvestris* clade with *M. dunensis* and *M. spartelensis* as a sister lineage. This pattern aligns with previous studies showing that 18S evolves slowly and is suitable for broad phylogenetic placement, whereas 28S evolves more rapidly and is highly informative for resolving closely related species^43,44^.

Phylogenetic reconstructions from concatenated PCGs, together with synteny comparisons, provided robust support for the species-level distinctiveness of *M. silvestris* and its broader placement within the genus. Consistent with patterns inferred from *nad5* markers^45^, our topologies recovered distinct tropical and temperate clades of root-knot nematodes, with *M. silvestris* occupying an intermediate position between these lineages.

Comparative synteny analysis showed that the PCG order in *M. silvestris* is largely conserved across *Meloidogyne* species and is more similar to *Pratylenchus vulnus* than to *Heterodera glycines*, consistent with previous studies indicating a closer evolutionary relationship between *Pratylenchus* and RKNs^46,47^. This suggests that mitochondrial gene order carries phylogenetic signals that complement sequence-based analyses and may help resolve relationships among plant-parasitic nematodes^48^. Within the limited dataset analysed in this study, Chromadorean nematodes appear to exhibit a more conserved mitochondrial gene order than the Enoplean taxa included. Nevertheless, the small number of Enoplea representatives prevents broader generalization across the clade.

The nearly complete mitochondrial genome of *M. silvestris* is a 15,048 bp circular molecule, encoding 12 protein-coding genes (*cox1-3*,* cob*,* nad1- 6*,* nad4L*,* atp6*), 18 tRNAs, and two rRNAs, consistent with other *Meloidogyne* species, which lack the *atp8* gene but otherwise exhibit a conserved PCG repertoire^49–51^. Genome size variation among root-knot nematodes is often driven by differences in the length and composition of non-coding regions^52^. Although mitogenome sizes of ~ 15 kb falls within the broader range reported for plant-parasitic nematodes, published mitochondrial genomes of *Meloidogyne* species are typically larger (> 17 kb). The relatively small size of the 15,048 bp mitogenome of *M. silvestris* therefore represents a deviation from the common size range for the genus and may reflect reduction in non-coding regions. The non-coding region is probably involved in regulatory functions analogous to the control regions described in *M. incognita* and *M. chitwoodi*^49^. Such regions often contain tandem repeats and secondary structures involved in replication, and their variation may contribute to intraspecific diversity^53^.

The strong A + T bias (76.6%) and pronounced AT (− 0.29) and GC (0.46) skews mirror patterns described for other chromadorean nematodes^48^. Such biases are thought to reflect asymmetric mutational processes during replication and transcription^54^. Consequently, amino acid alignments and models that account for compositional heterogeneity should be employed in phylogenomic analyses^55,56^.

Two gene overlaps (*nad5-atp6 and nad3-cob*) and a reduced tRNA complement (missing *trnA*,* trnH*,* trnQ*, and *trnR*) illustrate mitogenome compaction and may result from stop/start codon sharing^57^. To the best of our knowledge, no studies have reported a complete absence of nematode tRNAs; however, reduced or aberrant tRNAs are relatively common and may complicate genome annotation^58^. Functional compensation by nuclear-encoded tRNAs has been suggested in cases of true loss^59^. Manual inspection of predicted structures or experimental validation could help resolve whether these tRNAs are absent or simply undetected by computational tools.

## Conclusion

Across multiple lines of evidence, *Meloidogyne silvestris* populations showed striking uniformity, with concordant morphological, biochemical, and molecular data, including identical *cox1* and *cox2* barcodes, near-identical rDNA sequences, and a conserved mitogenomic architecture. These results confirm the taxonomic integrity of *M. silvestris* and demonstrate the power of integrative datasets for species delimitation in root-knot nematodes. The near-complete mitochondrial genome presented here expands genomic resources for the genus and provides a foundation for future studies of population structure, evolutionary dynamics, and the development of robust diagnostic markers.

## Supplementary Information

Supplementary Information 1.

## Data Availability

The supplementary dataset supporting this study is openly available in Zenodo at 10.5281/zenodo.20195346. The sequences generated in this study have been deposited in the GenBank database under the following accession numbers (in ascending order): OR826799 (18S rDNA), OR826800 (18S rDNA), OR827061 ( *cox1*), OR827062 (*cox1*), OR827063 (*cox**1*), OR827064 (*cox1*), OR831125 (28S rDNA), OR831126 (28S rDNA), and PZ371292 (complete mitochondrial genome of *Meloidogyne silvestris*). Additionally, the complete mitochondrial genome of *Meloidogyne hapla* was deposited in GenBank under accession number PZ371293.
