# Supplementary material for: Integrative taxonomy and mitogenome characterization of the root-knot nematode Meloidogyne silvestris
Source: Sci Rep. 2026 May 27;16:16439. doi: 10.1038/s41598-026-54669-9 (PMC13216338; doi:10.1038/s41598-026-54669-9)
Supplement: Supplementary file 1 — Supplementary Information 1. [file 41598_2026_54669_MOESM1_ESM.pdf]

Supplementary Information

Title: *Integrative taxonomy and mitogenome characterization of the root-knot nematode Meloidogyne silvestris*

Authors: Authors: *Justus Aisu*[1,2,3,4]*****, *Gerrit Karssen*[2], *Daniel Apolônio Silva De Oliveira*[1,2]

[1]Nematology Research Unit, Department of Biology, Ghent University, Ledeganckstraat 35, B-9000 Ghent, Belgium

[2]National Plant Protection Organization the Netherlands (NPPO-NL), Geertjesweg 15, 6706 EA Wageningen, The Netherlands.

[3]Geobotany and Botanical Garden, Martin Luther University Halle-Wittenberg, Halle (Saale), Germany

[4]German Centre of Integrative Biodiversity Research (iDiv) Halle-Jena-Leipzig, Leipzig, Germany

***Corresponding author: Justus Aisu, Email:** Justusaisu112@gmail.com

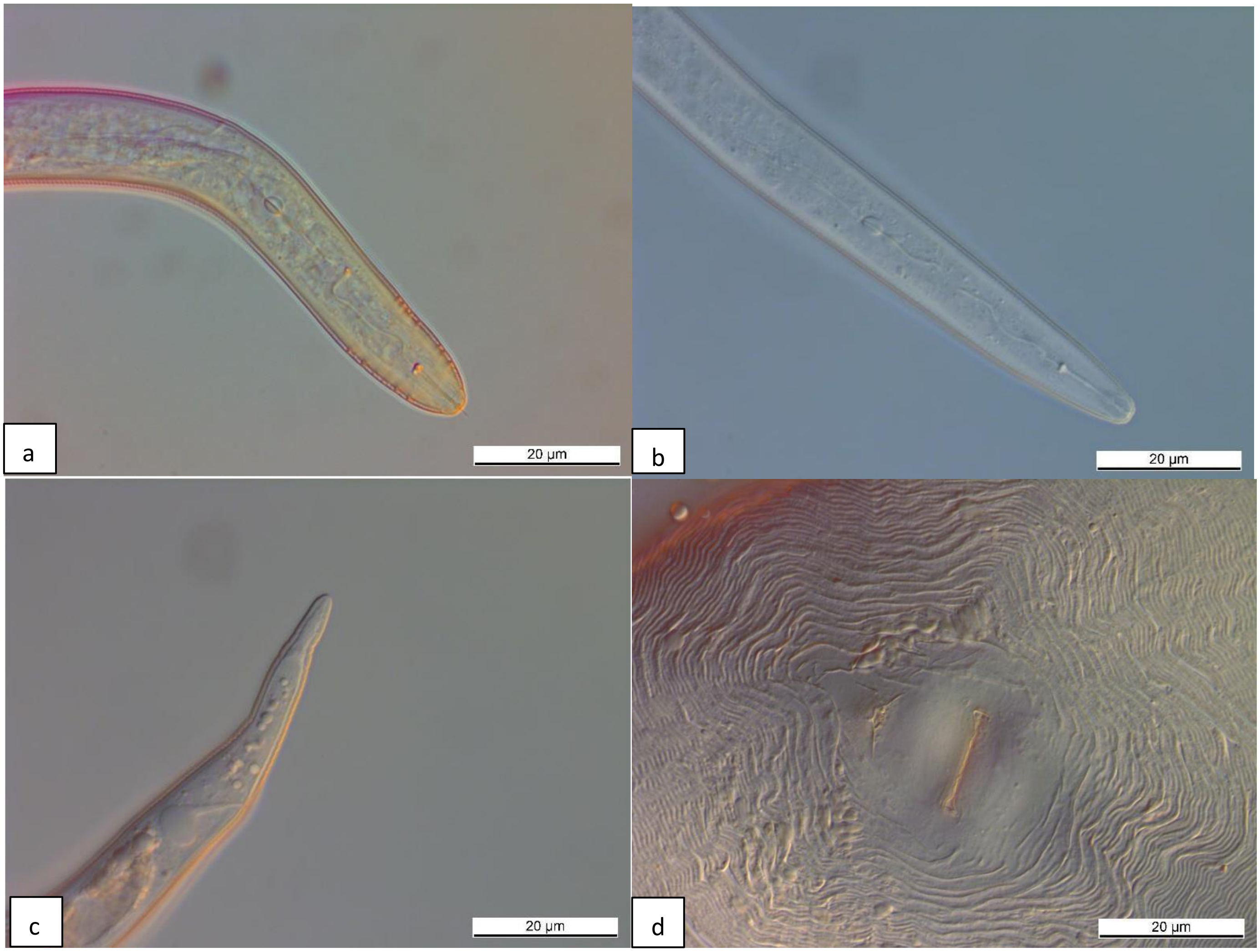

Fig. S1. Light micrographs of *Meloidogyne silvestris*. (a–b) Pharyngeal region of second-stage juveniles (J2). (c) Tail region of a second-stage juvenile (J2). (d) Perineal pattern of an adult female.

a)

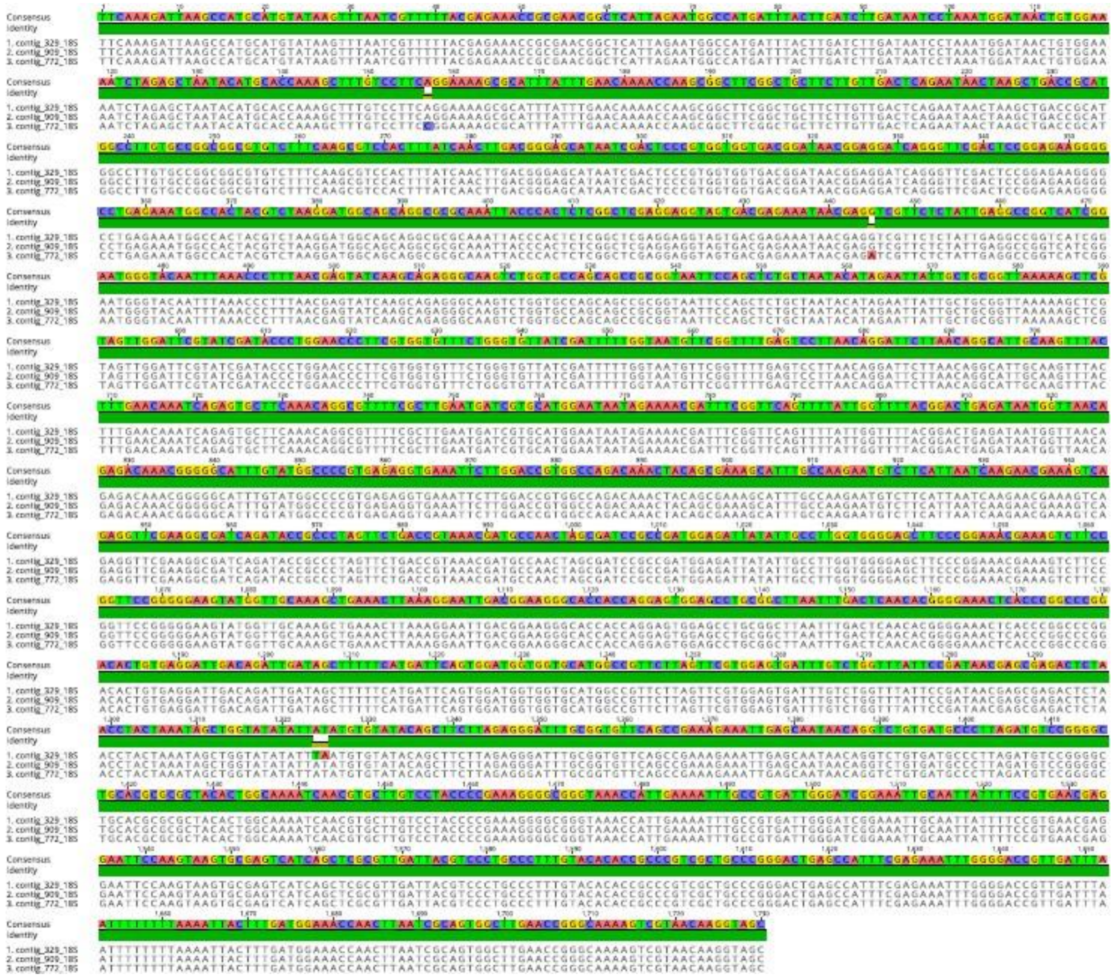

b)

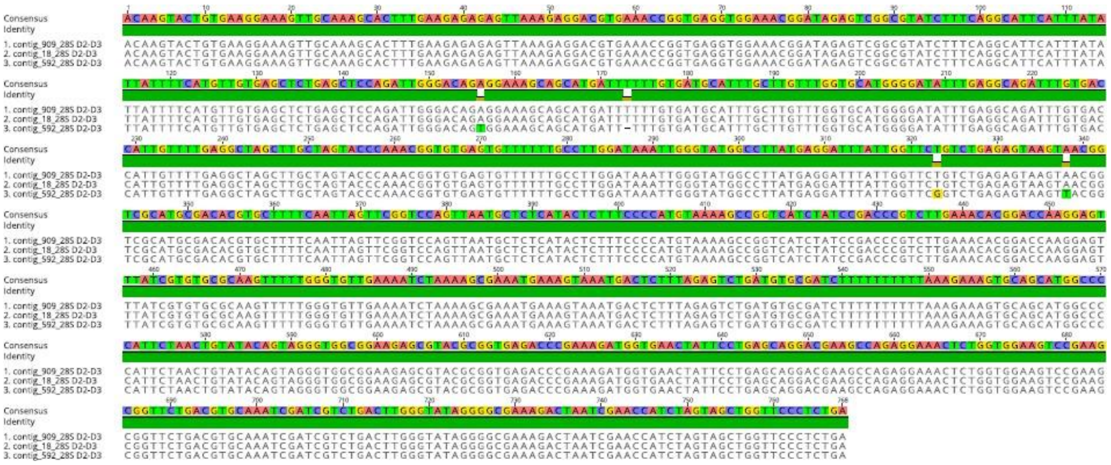

c)

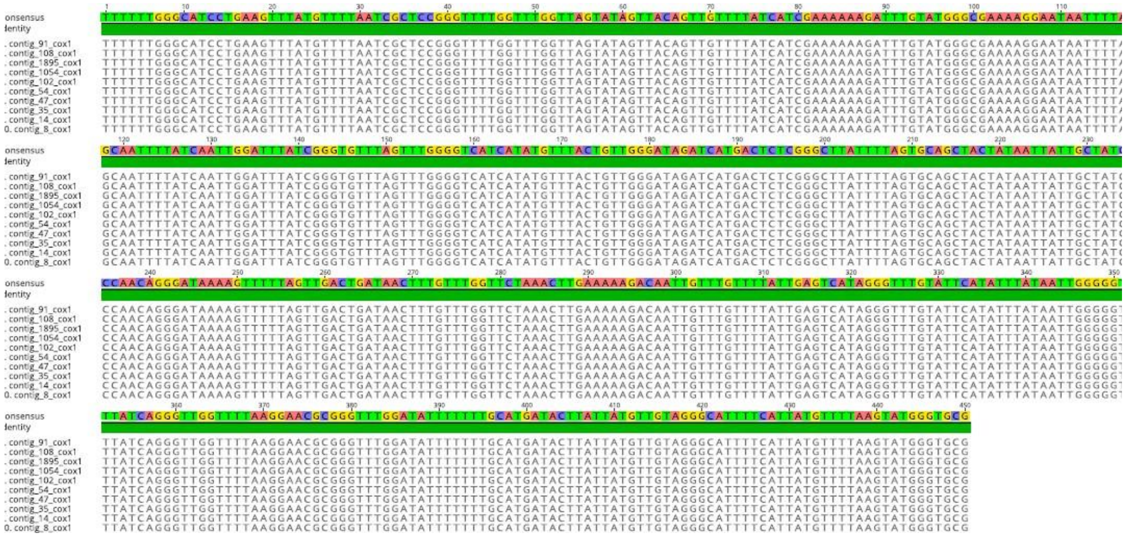

d)

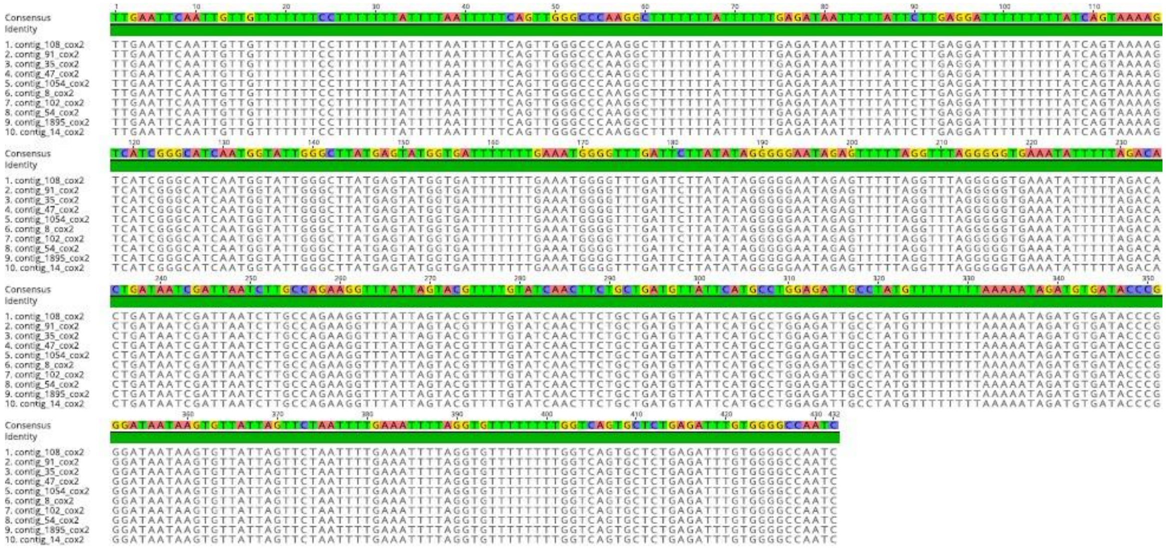

Fig. S2 | MAFFT alignment of *M. silvestris* 18S rDNA (a), 28S rDNA (b), cox1 (c), and cox2 (d) barcode sequences from specimens of two populations. The sequence alignments showed 99.9% and 99.7% similarity for the 18S and 28S regions, respectively, and 100% similarity for both cox1 and cox2 barcode regions.

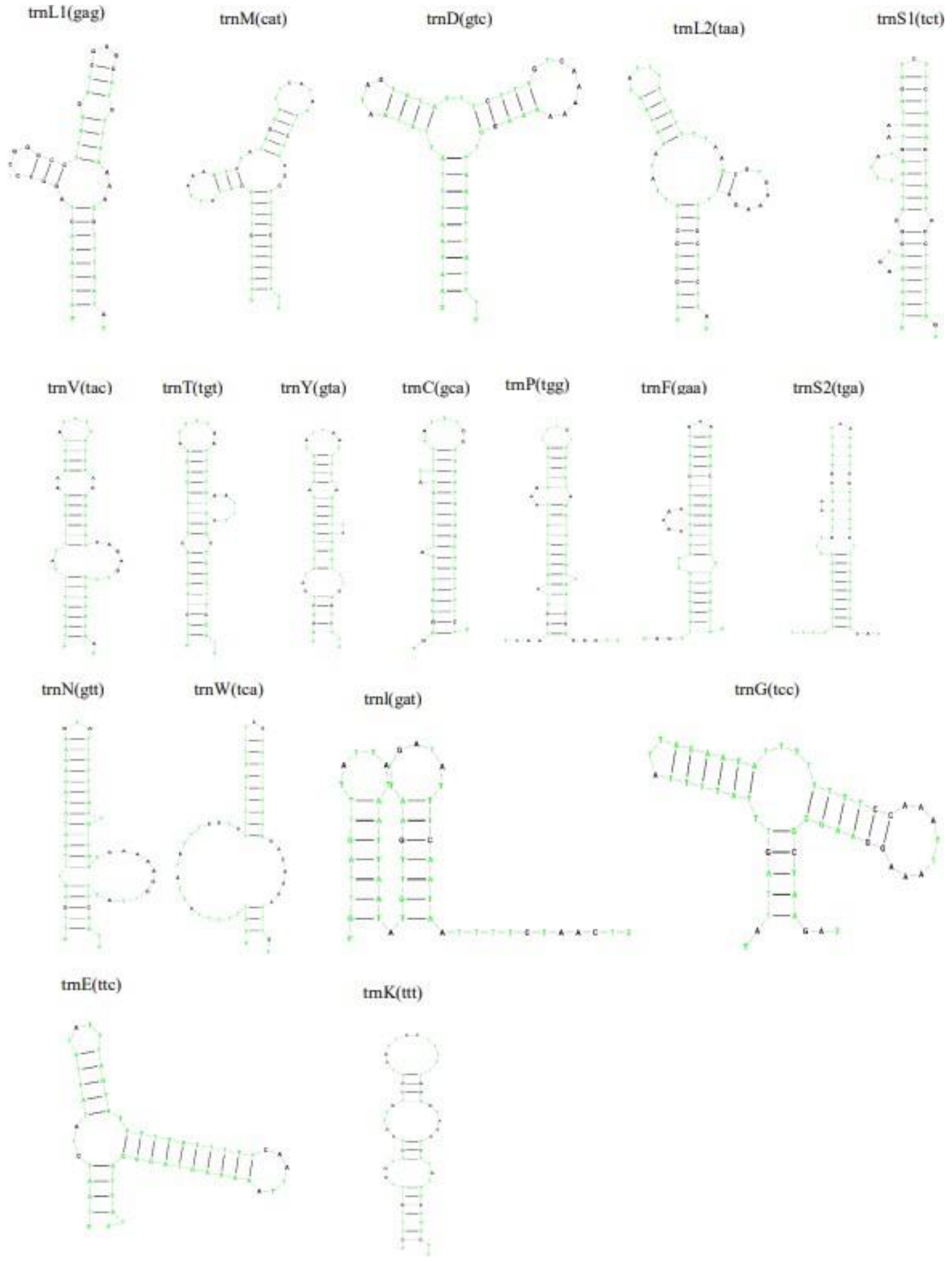

Fig. S3 | Predicted secondary structures of mitochondrial tRNAs from *M. silvestris,* visualized using the R2DT framework. Some tRNAs adopt typical stem–loop structures, whereas others exhibit reduced or modified arms.

Table S1. GenBank accession numbers for gene sequences of *Meloidogyne* species used in the present phylogenetic analysis

Species	**18S rRNA**	**D2-D3 of 28S rRNA**
M. abberans	MF278755	MF278754
M. africana	KY433422	KY433425
M. arabicida	HE667738	KF993624
M. ardenensis	AY593894	-
M. arenaria	AY942623	JX987332
M. artiellia	KC875391	AY150369
M. baetica	KP896296	AY150367
M. camelliae	JX912884	KF542869
M. chitwoodi	AY593884	AF435802
M. christiei	KR082316	KR082317
M. coffeicola	HE667739	-
M. cruciani	HE667740	-
M. daklakensis	-	KU243338
M. dunensis	EF612713	EF612712
M. duytsi	KJ636385	-
M. enterolobii	AY942629	KJ146862
M. ethiopica	KC551945	KF482372
M. exigua	AY942627	AF435795
M. fallax	AY593895	KC241969
M. floridensis	AY942621	-
M. graminicola	KF201168	KJ728847
M. graminis	JN241838	JN019326
M. hapla	AY942628	DQ145641
M. hispanica	HE667741	EU443607
M. ichinohei	KJ636350	EF029862
M. incognita	AY284621	JX100425
M. indica		MF680038
M. inornata	-	KF482374
M. izalcoensis	HE667743	KF993621
M. javanica	AY268121	KC953092
M. konaensis	HE667744	AF435797
M. kralli	KJ636370	-
M. lopezi	KF993645	KF993619
M. luci	LN713298	KF482371
M. mali	KJ636400	KF880398
M. maritima	EU669944	-
M. marylandi	JN241856	JN019333
M. microtyla	AF442198	-
M. minor	AY593899	JN628436

M. morocciensis	AY942632	KY882485
M. naasi	AY593900	KC241979
M. nataliei	MG821327	MG821326
M. oleae	MH011979	MH011963
M. oryzae	AY942631	-
M. paranaensis	AY942622	AF435799
M. partityla	KT825143	-
M. phaseoli	-	KY882487
M. salasi	-	KY962665
M. silvestris	EU570215	EU570214
M. silvestris	OR826799	OR831125
M. silvestris	OR826800	OR831126
M. spartelensis	KP896295	KP896293
M. spartinae	EF189177	-
M. thailandica	-	EU364890
M. trifoliophila	-	AF435801
P. penetrans	KJ934156	EU130860
P. vulnus	KC875389	EU130885

Table S2. List of mitochondrial genomes of nematode species and outgroup taxa included in the phylogenetic analyses, with corresponding GenBank accession numbers. Outgroup taxa (Arthropoda) were used to root the trees.

**Species names**	**GenBank accession number**
Ascaridia galli	JX624728
Ascaris lumbricoides	HQ704900
Ascaris suum	HQ704901
Baylisascaris procyonis	JF951366
Bursaphelenchus mucronatus	GU177865
Bursaphelenchus xylophilus	GQ332424
Caenorhabditis briggsae	NC_009885
Caenorhabditis elegans	NC_001328
Cucullanus robustus	GQ332426
Enterobius vermicularis	EU281143
Haemonchus contortus	EU346694
Heterodera glycines	HM640930
Heterorhabditis bacteriophora	EF043402
Hexamermis agrotis	EF368011
Limulus polyphemus (Outgroup)	NC_003057

Lithobius forficatus (Outgroup)	NC_002629
Meloidogyne arenaria	KP202350
Meloidogyne chitwoodi	KJ476150
Meloidogyne enterolobii	KP202351
Meloidogyne graminicola	NC024275
Meloidogyne hapla	PZ371293
Meloidogyne incognita	KJ476151
Meloidogyne javanica	KP202352
Meloidogyne oryzae	MK507908
Meloidogyne silvestris	PZ371292
Pratylenchus vulnus	GQ332425
Pristionchus pacificus	JF414117
Steinernema carpocapsae	AY591323
Strongyloides stercoralis	AJ558163
Thaumamermis cosgrovei	DQ520857
Trichinella spiralis	AF293969
Trichuris suis	GU070737
Wellcomia siamensis	GQ332427
Xiphinema americanum	AY382608
Xiphinema paciatum	NC033870
